# Thermodynamic and Structural Behavior of α‐Galactosylceramide and C6‐Functionalized α‐GalCer in 2D Layers at the Air–Liquid Interface

**DOI:** 10.1002/cbic.201900491

**Published:** 2019-11-07

**Authors:** Gerald Brezesinski, Adam D. J. Calow, Claney L. Pereira, Peter H. Seeberger

**Affiliations:** ^1^ Max Planck Institute of Colloids and Interfaces Biomolecular Systems Department Am Mühlenberg 1 14476 Potsdam Germany

**Keywords:** α-galactosylceramide, immunotherapy, monolayers, structural properties

## Abstract

α‐Galactosylceramide (α‐GalCer; KRN7000) is a ligand for the glycoprotein CD1d that presents lipid antigens to natural killer T cells. Therefore, KRN7000 as well as some modified versions thereof have been widely investigated as part of novel immunotherapies. To examine the impact of structural modification, we investigated KRN7000 and C6‐modified KRN7000 at the air–liquid interface using monolayer isotherms, BAM, IRRAS, GIXD, and TRXF. The amino group has no influence on the highly ordered sub‐gel structures found at lateral pressures relevant for biological membranes. Neither lateral compression nor the protonation state of the amino group has a measurable effect on the lattice structure, which is defined by strong and rigid intermolecular hydrogen bonds. However, the first‐order phase transition found for the C6‐functionalized α‐GalCer is connected with an extraordinary surface‐inhibited nucleation. Our study demonstrates that KRN7000 can be functionalized at C6 without significantly changing the structural properties.

## Graphical abstract

**Robust under pressure**: α‐Galactosylceramide (KRN7000) and analogues thereof are used in novel immunotherapies, but little is known about the influence of structural modifications on the physical properties of the conjugates. By investigating 2D monolayers at the air–liquid interface, this study shows that KRN7000 can be modified at C6 without changing its highly ordered sub‐gel structures.

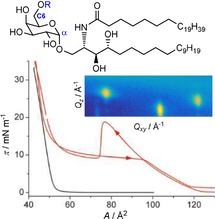

## Introduction

**Figure 1:**
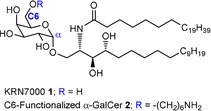
KRN7000 **1** and C6‐functionalized α‐GalCer **2**.

In the early 1990s, several glycosphingolipids from the marine sponge *Agelas mauritianus* were isolated.^1^, ^2^ Structural elucidation revealed that the glycosphingolipids consisted of ceramides α‐glycosidically linked to d‐galactose (e.g., **1**). Subsequent structure–activity work found the α‐galactosyl*ceramide* (α‐GalCer) KRN7000 **1** to be a highly potent tumor growth inhibitor in mice (Figure 1).^3^ The therapeutic properties and applications of KRN7000 have been the subject of many reviews.^4^, ^5^, ^6^ Glycosphingolipids continue to be of interest due to their biological significance and potential applications.^7^, ^8^, ^9^, ^10^

KRN7000 serves as ligand for the glycoprotein CD1d.^11^ CD1d is expressed on the surface of antigen presenting cells and is involved in presenting lipids antigens to natural killer T cells. This event leads to the production of cytokines, resulting in a Th1 or Th2 immune response. Due to the immunological significance of NKT cells, KRN7000 and other analogues have been investigated in the context of novel immunotherapies.^12^

Work toward synthetic vaccines to protect against a variety of infectious diseases^13^, ^14^ in our laboratory^15^ as well as others^16^ have illustrated the utility of KRN7000 to self‐adjuvant conjugate vaccines.^17^ Conjugation of α‐GalCer **2** to the capsular polysaccharide of *Streptococcus pneumoniae* serotype 4 induced long‐term protection against serotype 4 in murine models. Serotype 4 polysaccharide was conjugated to α‐GalCer **2** via an alkyl amino linker at the C6 hydroxy group of galactose. KRN7000 can be functionalized at specific sites. Functionalization at the C6 position, either by the introduction of small molecules^18^ or spacers to allow for late‐stage conjugation, is possible without significantly inhibiting effective CD1d presentation.^19^ Biological and crystallographic data of murine CD1d^20^ and human CD1d, with and without KRN700 binding support this notion.^21^

Monolayers of amphiphilic molecules are meaningful models of one half of biomembranes to understand structure formation in 2D as well as interactions of biologically important molecules, dissolved in the subphase, with the membrane surface. Early work suffered from the lack of sophisticated surface‐sensitive tools to investigate liquid interfaces with molecular and microscopic resolution. Meanwhile, numerous techniques, like Brewster angle microscopy, X‐ray and neutron scattering, infrared reflection‐absorption spectroscopy (IRRAS), and nonlinear optical spectroscopy, are available. Langmuir monolayers can be easily manipulated by simple mechanical compression (increasing the density of molecules at otherwise identical conditions).^22^, ^23^, ^24^, ^25^ However, Langmuir monolayers suffer drawbacks as the phospholipid systems are metastable, and transmembrane processes as well as the incorporation of proteins cannot be investigated.

The first step in Langmuir monolayer research is recording of surface pressure (*π*)–molecular area (*A*) isotherms. Phase transitions, triggered by changing the packing density of the molecules, can be easily identified. The first‐order phase transition from the disordered LE phase to any ordered condensed phase is characterized by a plateau region in which both phases coexist. The hydrophobic chains are in an ordered (all‐*trans*) state, and the monolayer structure can be studied on an Angstrom scale by grazing incidence X‐ray diffraction (GIXD).^26^, ^27^, ^28^, ^29^

Little is known about the influence that structural modifications impart on the physical properties of the glycolipid KRN7000 **1** and C6‐functionalized α‐GalCer **2**. We investigated 2D monolayers of these glycolipids at the air–liquid interface using different surface‐sensitive methods, and examined the impact of the C6 functionalization on the physical properties and phase behavior of KRN7000.

## Results and Discussion

**Scheme 1:**
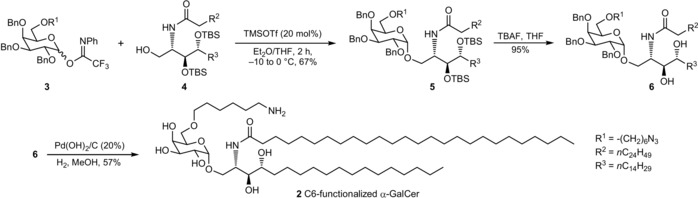
Synthesis of C6‐functionalized α‐GalCer.

KRN7000 **1** and its analogues have been prepared via different synthetic routes.^30^, ^31^, ^32^, ^33^ Protected α‐GalCer **5** can be accessed through the facile union of galactosyl imidate **3** and ceramide **4** (67 % yield, Scheme 1).^34^ Without participating groups to direct the glycosylation, ethereal solvents ensured the exclusive formation of the α‐anomer **5**.^35^ Two‐step deprotection relied on desilylation (TBS cleavage) of **5** by treatment with TBAF (95 %) before palladium‐catalyzed hydrogenation, which allowed for the isolation of 57 % of the C6‐functionalized α‐GalCer **2**.

**Figure 2:**
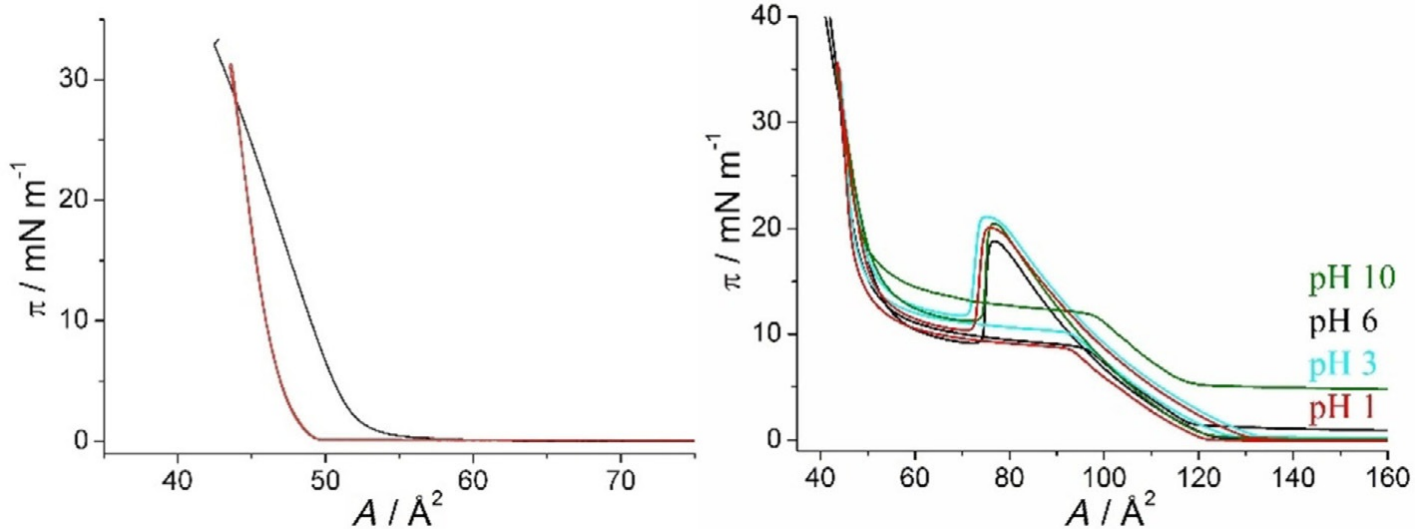
Left: Pressure–area (*π*–*A*) isotherms of KRN7000 **1** monolayers on water at 20 °C (black) and at 37 °C (red). Right: *π*–*A* isotherms of the C6‐functionalized α‐GalCer **2** monolayers on subphases with different pH values (indicated) at 20 °C.

The *π*–*A* isotherms of KRN7000 **1** and the C6‐functionalized α‐GalCer **2** are markedly different (Figure 2). The isotherm of KRN700 **1** is typical for a monolayer which does not form a liquid‐expanded (LE) phase (Figure 1) up to temperatures of 37 °C. The gas‐analogous state transforms during compression directly into a condensed (LC) state (re‐sublimation process) at practically zero lateral pressure. Apparently, the compressibility at 20 °C is higher than at 37 °C, a temperature at which the film is almost incompressible. However, the reason for this apparent discrepancy must be the different spreading behavior at the two temperatures. Likely, islands of the LC phase formed during spreading must be much larger at lower temperature. Therefore, the defect density in the layer is much higher and difficult to be removed during compression. At higher temperature, the film is more homogeneous.

The *π*–*A* isotherms of C6‐functionalized α‐GalCer **2** have a lift‐off point around 120 Å^2^ indicating the existence of a liquid‐expanded (LE) phase at low lateral pressures. The transition into the LC phase is characterized by a drastically hindered nucleation observed by the huge hump in the isotherms. The surface inhibited nucleation needs an excessive over‐compression (supersaturation of the LE phase) for starting the nucleation process. The nucleation rate is high and the growth rate low leading to many small domains (BAM image in Figure S1). Reducing the compression speed does not change the shape of the isotherms (data not shown). Increasing temperature shifts the transition to higher lateral pressures but does not change the surprising behavior. The highest pressure needed for starting the nucleation is always approximately 11 mN m^−1^ higher than the corresponding transition pressure *π*_t_. The observed difference (Figure S2 in the Supporting Information) increases only by 0.8 mN m^−1^ if the temperature is increased by 10 K. Therefore, the compression curves are far from equilibrium and cannot be used for the thermodynamic analysis by the Clausius–Clapeyron equation. The pH has practically no influence on this behavior even if the terminal amino group should be protonated and therefore positively charged at low pH values and uncharged at high values (p*K*_a_≈10.6).^36^ The only influence of pH is observed in the decompression curve at pH 10. Zero pressure is not achieved even at large molecular areas what could indicate that denser areas of a non‐homogeneous film are still in contact with the Wilhelmy plate.

**Figure 3:**
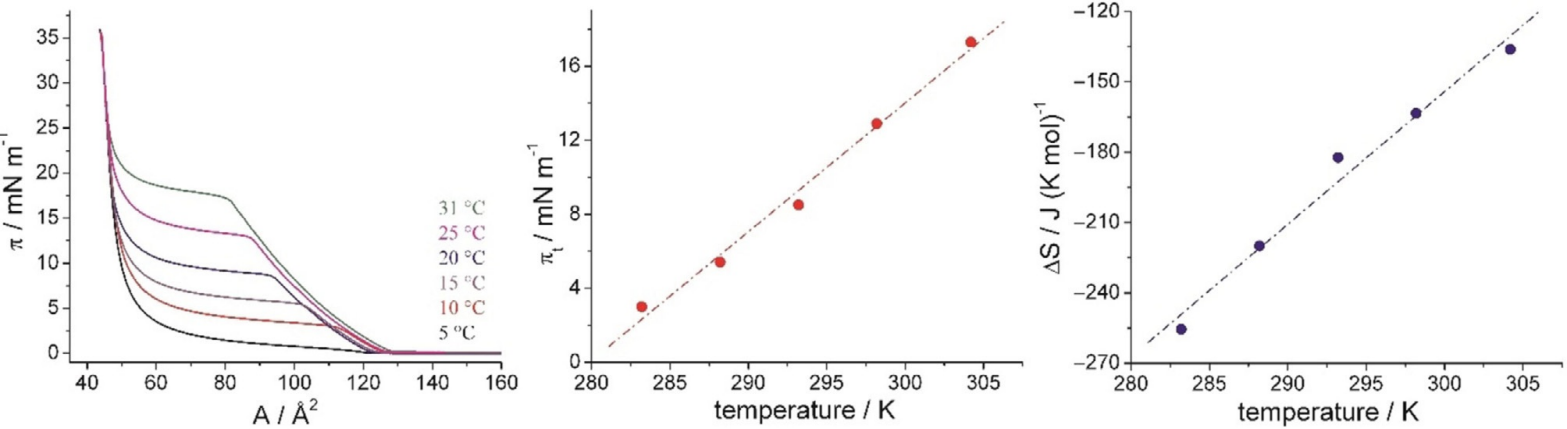
Left: Decompression *π*–*A* isotherms of the C6‐functionalized α‐GalCer **2** monolayers on pH 1 subphase taken at different temperatures (indicated). Middle: Temperature dependence of the main phase transition pressure *π*_t_. Right: Temperature dependence of the entropy change Δ*S* at the LE/LC phase transition.

The decompression curves at low pH values are equilibrium ones and can be used for the thermodynamic analysis. The influence of temperature on the decompression curves at pH 1 is shown in Figure 3.

The phase transition pressure (*π*_t_) is determined from the kink in the *π*–*A* isotherms, which indicates the onset of the first‐order LE–LC phase transition. Evaluating the temperature dependence of the phase transition pressure gives access to the transition entropy. The slope d*π*_t_/d*T* of the linear fit to the experimental data amounts to 0.696 mN m^−1^ K^−1^. The entropy change Δ*S* of the phase transition was calculated using the two‐dimensional Clausius–Clapeyron equation [Eq. (1)]:

(1) $$\Delta S=\left(A{}_{c}-A{}_{e}\right)\frac{d\pi {}_{t}}{dT}$$

with *A*_e_ as the molecular area at the onset of the phase transition at the surface pressure *π*_t_ and *A*_c_ as the area of the corresponding condensed phase at the same lateral pressure.^37^ The temperature dependence of the entropy change Δ*S* is presented in Figure 3, right. The main phase transition at compression of amphiphilic monolayers is an exothermic process, therefore, negative Δ*S* values are obtained. Extrapolation of Δ*S* to zero yields the critical temperature *T*_c_ of 327.2 K (54.0 °C), above which the monolayer cannot be compressed into the condensed state. On the other hand, the temperature *T*_0_ is 279.8 K (6.6 °C). Below this temperature, no LE phase is existing and the gas‐analogous phase transforms directly into the LC phase (re‐sublimation process) as observed for KRN7000 **1**.

**Figure 4:**
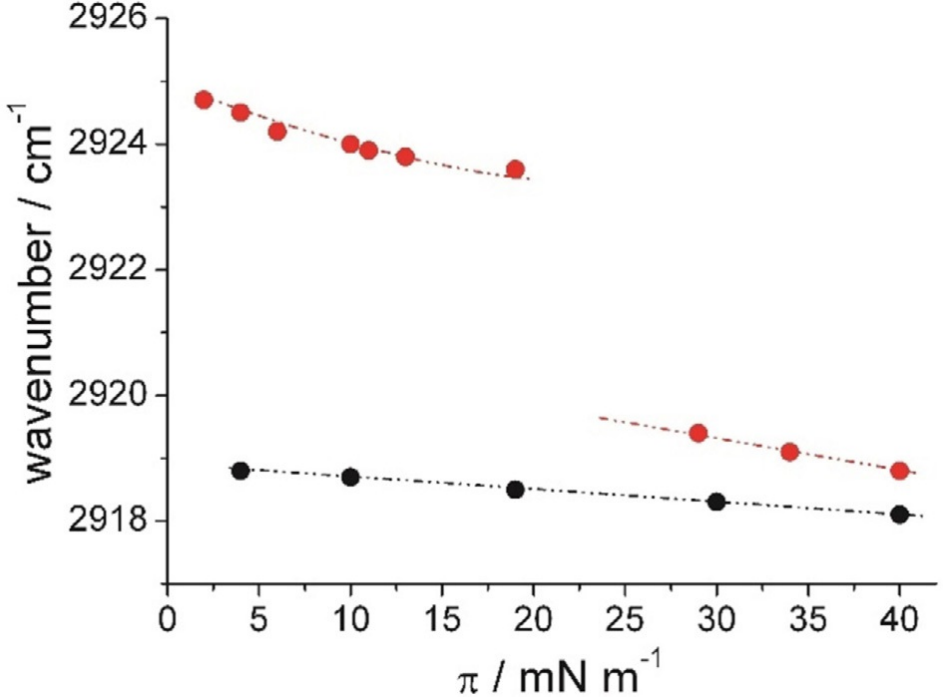
Wavenumbers of the asymmetric CH_2_ stretching vibration taken at different lateral pressures *π* along the isotherms of KRN7000 (black) and the C6‐functionalized α‐GalCer (red).

IRRAS supports the conclusions drawn from the isotherm experiments. The CH_2_ stretching vibrations are sensitive to the phase state of the alkyl chains. The wavenumbers *ν*_asym_ obtained in IRRAS experiments along the isotherms of KRN7000 **1** and the C6‐functionalized α‐GalCer **2** are presented in Figure 4 (IR spectra are presented in Figures S3 and S4). The values between 2926 and 2924 cm^−1^ indicate substantial gauche conformers. The first‐order phase transition in the C6‐functionalized α‐GalCer **2** monolayers occurs above 20 mN m^−1^, in agreement with the compression isotherm. Please note that the IRRAS experiments are performed at constant pressure. Each experiment at fixed pressure takes approximately 10 minutes. This means that even keeping the monolayer in the over‐compressed state for a longer time does not induce the phase transition below 20 mN m^−1^. The LE–LC phase transition can also be observed in the intensity of the OH band. The increase in OH‐band intensity (Figure S3) indicates an increase in the effective thickness/density of the monolayer. In contrast, the intensity of the OH band along the isotherm of KRN7000 is practically constant (Figure S4) indicating a constant thickness and only marginal changes in the packing density (removing defects upon compression).

In the LC phase, low wavenumbers between 2919 and 2918 cm^−1^ are typical for a tightly packed condensed state. The differences between the wavenumbers of KRN7000 **1** and the C6‐functionalized α‐GalCer **2** in the condensed state are only marginal.

**Figure 5:**
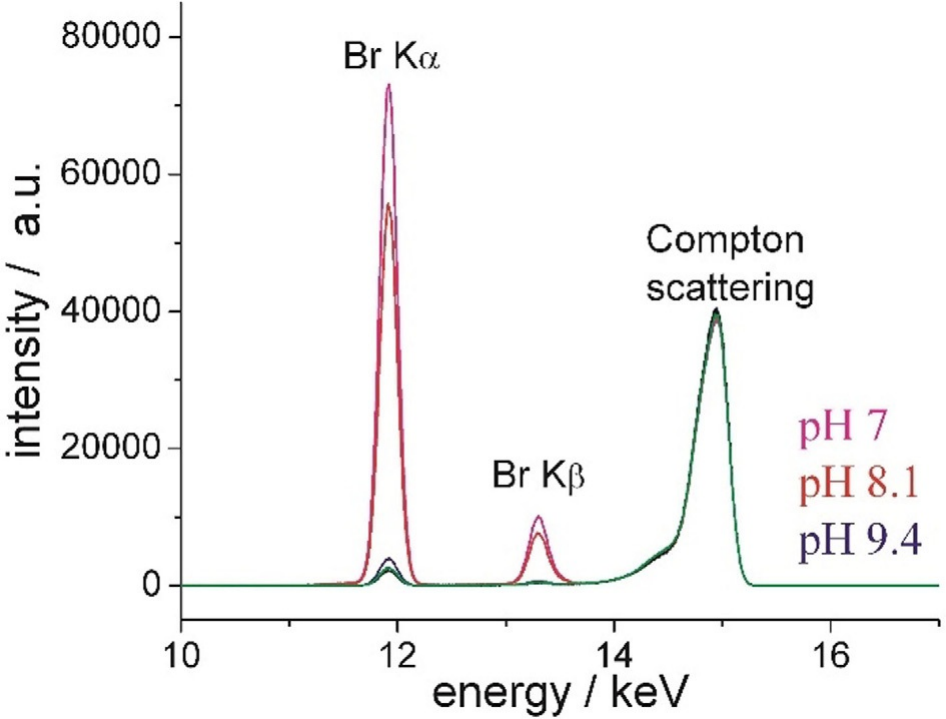
Selected part with the most interesting fluorescence lines of the X‐ray fluorescence spectra of the C6‐functionalized α‐GalCer **2** at a surface pressure of *π*=20 mN m^−1^ on subphases at different pH values (indicated) containing 1 mm of the used counterion Br^−^. For comparison, the spectra of the bare subphase at pH 7 (black) and of KRN7000 **1** (green) on this subphase are shown.

To determine the protonation state of the amino group in monolayers of the C6‐functionalized α‐GalCer **2**, TRXF measurements were performed on subphases with pH values between 7.0 and 9.4 around the expected p*K*_a_ value (Figure 5). The positively charged amino group attracts negatively charged counter‐ions from the subphase in order to form the electrical double layer (EDL). In the present case, the anion Br^−^ is used in a concentration of 1 mm. The experiments show clearly a decreasing intensity of the Br fluorescence lines (Kα at 11.92 keV and Kβ at 13.29 keV) with increasing pH validating a p*K*_a_ value of ≈9 for the terminal amino group. The fluorescence intensity of the Br at pH 9.4 is only double of that of the background (bare subphase). KRN7000 **1** as an uncharged layer does not show any fluorescence intensity above the corresponding background values.

The p*K*_a_ value of the amino group of the C6‐functionalized α‐GalCer **2** can be determined with high precision using a subphase buffer with only one type of anion to avoid any competition of anions for interactions with the charged head groups.^38^

**Figure 6:**
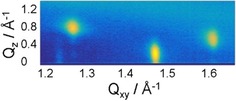
Contour plots (diffracted intensities plotted as contour lines of equal intensity vs. the in‐plane component *Q_xy_* and the out‐of‐plane component *Q_z_* of the scattering vector) of the C6‐functionalized α‐GalCer **2** in the wide‐angle region. Experiments are performed on water at 20 mN m^−1^ and 20 °C.

Grazing incidence X‐ray diffraction (GIXD) was employed for defining the structural order of the monolayers down to an ångström level. Interestingly, both compounds, feature very different thermodynamic properties and exhibit the same structural properties. The structural differences observed on different subphases (salts, pH) are too marginal to be discussed. The GIXD data obtained from both compounds in the condensed phase revealed that the lateral order of the chains is defined by three strong diffraction peaks (Figure 6) measured in the wide‐angle region.

The diffraction pattern practically does not change upon compression. This indicates a “frozen” oblique lattice structure. The structure is characterized by strongly tilted alkyl chains and a very small cross‐sectional area *A*_0_ indicating extremely tight packing with no chain rotation. Comparing the two compounds shows the similarity of the condensed phase structures: KRN7000 **1** has a chain tilt of 34.2° and a cross‐sectional chain area *A*_0_ of 18.4 Å^2^ whereas the C6‐functionalized α‐GalCer **2** has a slightly lower chain tilt of 33.9° and a slightly larger *A*_0_ of 18.6 Å^2^ leading to the same in‐plane molecular area of ≈44.5 Å^2^ in agreement with the values determined from the area–pressure isotherms.

**Figure 7:**
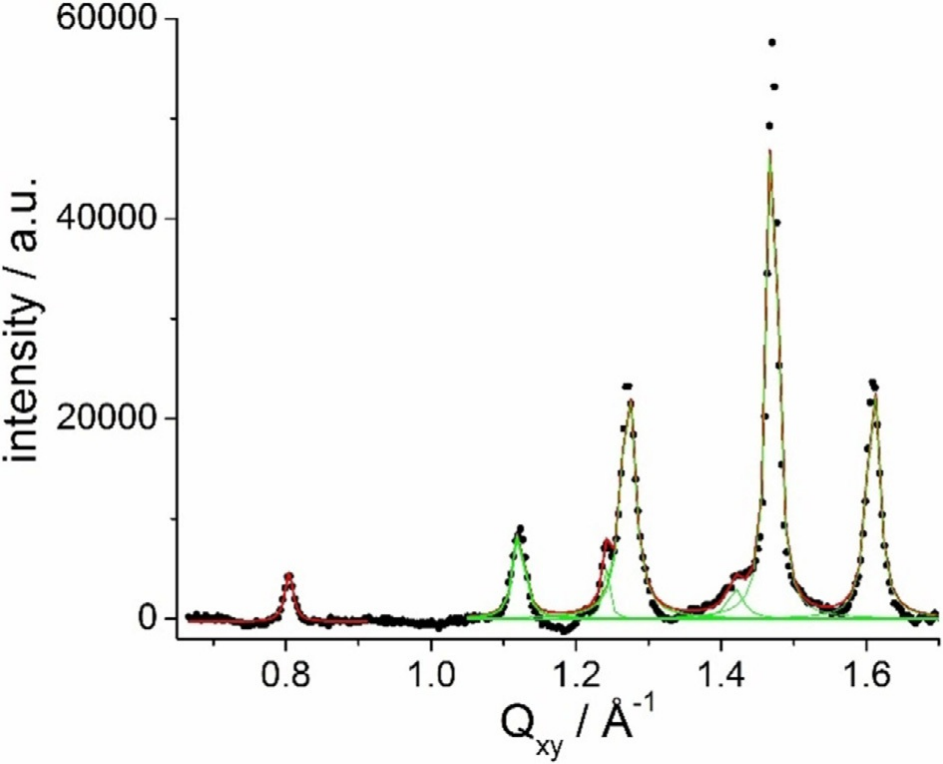
Bragg peaks (scattered intensity integrated over the whole *Q_z_* window of the used detector versus the *Q_xy_* component of the scattering vector) of KRN7000 on 1 mm CaCl_2_ at 20 mN m^−1^, and 20 °C.

Interestingly, additional Bragg peaks can be seen in the GIXD patterns of both compounds (Figure 7). These additional peaks indicate a higher degree of order involving whole molecules. The indexing of those additional Bragg peaks is based on a larger supercell induced most probably by strong intermolecular hydrogen bonds established between the head groups, similar to the previously reported monolayer structures of other glycolipids and a GPI fragment.^39^, ^40^ The supercell of this sub‐gel phase must be commensurate with the hydrocarbon chain lattice. Using as unit cell parameters *a′*=2×a_chains_=9.0 Å, *b′*=2×b_chains_=10.4 Å, and *γ*=108.5°, the following Bragg peaks can be determined and compared with the experimentally observed ones in Table 1.

**Table 1:** Experimentally observed and calculated Bragg peak positions for KRN7000 on 1 mm CaCl_2_ at 20 mN m^−1^ and 20 °C.

Miller indices	(0 1)	(1 0)	(1 −1)	(1 1)	(1 −2)	(0 2)	(2 −1)	(2 0)	(2 −2)
(*h k*)	(0 −1)	(−1 0)	(−1 1)	(−1 −1)	(‐1 2)	(0 −2)	(−2 1)	(−2 0)	(−2 2)
*Q_xy_* [Å^−1^] calcd.	0.637	0.736	0.806	1.116	1.253	1.274	1.407	1.472	1.613
*Q_xy_* [Å^−1^] exptl.			0.804	1.121	1.241	1.272	1.418	1.472	1.612

The calculated area of 88.8 Å^2^ corresponds to that of two molecules. The head group order based on a rigid hydrogen‐bond network, which is practically uncompressible, dictates the packing order of the chains and is responsible for the high tilt angle.

**Figure 8:**
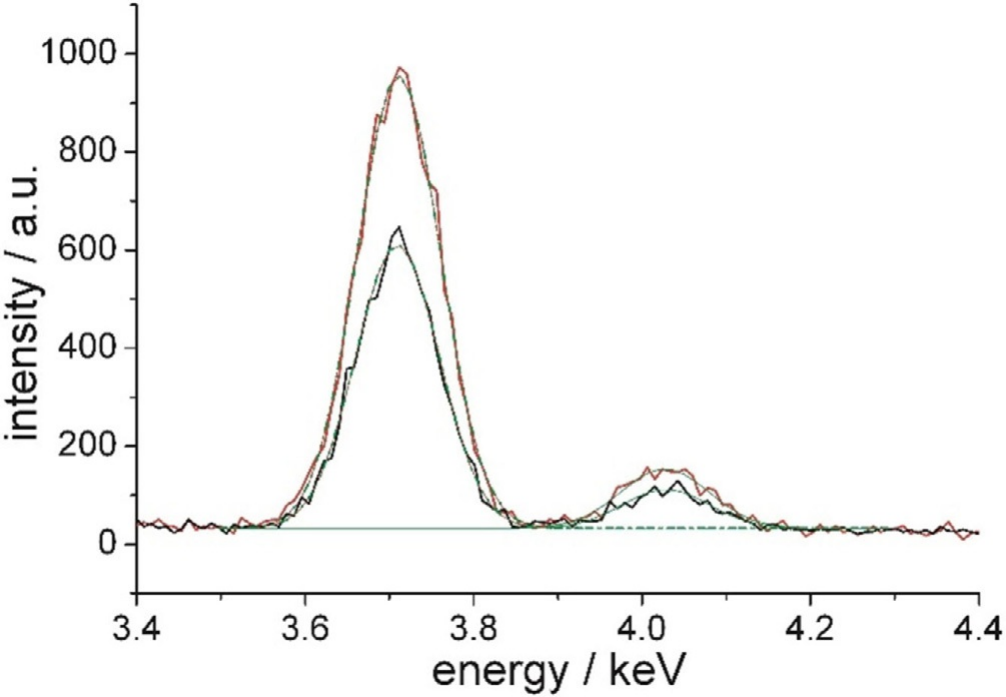
Selected part of the X‐ray fluorescence spectra of KRN7000 (red) on a 1 mm CaCl_2_ subphase at a surface pressure of *π*=20 mN m^−1^ and of the same subphase with no KRN7000 monolayer on top (black) with the Kα (3.69 keV) and Kβ (4.01 keV) fluorescence lines of calcium. Gaussian functions (green) have been fitted to the corresponding experimental curves.

Selective interactions between ions and charge‐neutral glycolipids can have far‐reaching consequences in many biological events. Earlier, preferential interactions of ions with uncharged glycolipid Langmuir monolayers at the air–water interface have been directly quantified.^40^ All monolayers featuring head group order (formation of sub‐gel phases) exhibited preferential interactions with certain ions suggesting that the defined structural motifs of these highly ordered surfaces are responsible for the observed ion selectivity. Because glycolipid KRN7000 is charge neutral and forms highly ordered monolayer structures, first TRXF experiments were carried out to quantify preferential interactions of calcium with the KRN7000 monolayer. In the above described experiments, it has been shown that Br^−^ ions are not attracted by the ordered head groups. Nevertheless, experiments with calcium ions indicate another behavior. The excess of calcium is deduced from the intensity of its element‐characteristic X‐ray fluorescence (integrated Kα and Kβ lines) normalized to the intensity of the evanescent standing wave *Φ* at the location of the ion.^38^ The intensity of the standing wave decays approximately exponentially with the depth [Eq. (2)]:

(2) $$\Phi \left(\theta ,z=d{}_{\mathrm{HC}}\right)\approx \Phi \left(\theta ,z=0\right)e{}^{-d{}_{\mathrm{HC}}/\Lambda }$$

with *Λ*≈7 nm as the decay length for the used beam energy of 15 keV and incident angle of 0.07°, and *d*_HC_ as the thickness of the hydrophobic part of the monolayer. *I*_ex_=(*I*–*I*_0_)/*I*_0_ is the relative change in the measured intensity *I* with respect to the intensity *I*_0_ expected in the absence of a monolayer. *I*_0_ is obtained by measuring the bare ion containing aqueous subphase, and taking into account the reduction of the intensity at the place where the ion is located in presence of the monolayer (Figure 8).

Ion excess can be determined on an absolute scale (absolute number of excess ions per nm^2^) by using charged amphiphiles and ions with no noticeable mutual complexing ability. For cations, highly charged (charge density of ≈0.64 C m^−2^) Langmuir monolayers of long‐chain alkylsulfates (behenylsulfate, BS) have been used successfully.^41^ The head groups of these monolayers are completely deprotonated under the used conditions. The maximum packing density, observed in pressure‐area isotherms, is close to 25 Å^2^⋅molecule^−1^.

The thickness of the hydrophobic part *d*_HC_ of the monolayer can be estimated by Equation (3):

(3) $$d{}_{\mathrm{HC}}=l{}_{\max}\cos\left(t\right)$$

with *l*_max_ as the maximum length of a stretched alkyl chain with *n* CH_2_ groups *l*_max_=(*n*×1.26+1.5) Å.^42^ With the tilt angle of the chains *t* (see above) determined by GIXD, 28 Å is calculated for KRN7000 and 23 Å for BS. The excess intensity *I*_ex_ amounts to 0.604 for KRN7000 and 28.419 for BS. With one Ca^2+^ ion per 50 Å^2^ (compressed BS layer), the experimentally determined excess for the KRN7000 layer corresponds to an area per adsorbed calcium ion of ≈24 nm^2^ or one calcium ion per approximately 53 glycolipids. The present results demonstrate imposingly that the uncharged hydrophilic surface of KRN7000 can attract calcium ions.

## Conclusion

Most surprising is the formation of condensed phases in monolayers of molecules with such a large chain mismatch. Additional experiments, which are beyond the scope of this paper, are required to develop a reasonable model. Amino groups linked via an alkyl spacer to the galactose of KRN7000 have no influence on the highly ordered sub‐gel structures found at lateral pressures relevant for biological membranes (30–35 mN m^−1^).^43^ Neither lateral compression to higher surface pressures nor the pH of the subphase has a measurable effect on the lattice structure. The extremely small cross‐sectional chain area indicates very tight packing (herringbone mode) with no rotational freedom. The supercell indicates ordering of entire molecules which is most likely induced by strong and rigid intermolecular hydrogen bonds between the galactosylceramide head groups dictating the packing of the chains. The amino group is decoupled from the strongly interacting head groups and can be even positively charged, as proved by TRXF, with no effect on the monolayer structure. However, the thermodynamic monolayer properties are clearly influenced. The C6‐functionalized α‐GalCer **2** forms a liquid‐like phase (LE) at low lateral pressures in contrast to parent compound KRN7000 **1**. The first‐order phase transition to the highly ordered condensed phase is connected with an extraordinary surface inhibited nucleation (supersaturation of the LE phase). Most likely, the linker with the amino groups bends toward the interface in the disordered LE phase. Therefore, over‐compression of the layer is needed for the formation of this highly ordered sub‐gel structure, and the isotherms are similar at all subphase pH values.

This study demonstrates that KRN7000 can be functionalized at specific sites without significantly changing the structural properties at biologically relevant lateral pressures in accordance with biological data.

## Experimental Section

**Materials and monolayer experiments**: For the monolayer experiments, the glycolipid (e.g., **1** or **2**) was dissolved in chloroform to a concentration of 1 mm. The solutions were spread by a micro‐syringe onto different subphases. The *π*–*A* isotherms were recorded on a computer‐interfaced Langmuir trough (R&K, Potsdam, Germany) equipped with a Wilhelmy balance system after a 10 min wait to ensure complete solvent evaporation. The surface tension of water *σ*_w_, measured with a filter paper Wilhelmy plate, decreases with increasing concentration of amphiphilic molecules at the surface (*σ*_F_). The surface pressure *π*=*σ*_w_−*σ*_F_ is plotted versus the molecular area A. A constant temperature was adjusted and stabilized using a recirculation cooler. The compression speed of the film was 5 Å^2^ molecule^−1^ min^−1^. All isotherms were measured at least twice for reproducibility.

**Brewster angle microscopy (BAM)**: The morphology of the monolayer was imaged with a Brewster angle microscope, model BAM2plus from NanoFilm Technologie (Göttingen, Germany), equipped with a miniature film balance from NIMA Technology (Coventry, UK), both mounted on an antivibration table. The microscope was equipped with a frequency‐doubled Nd:YAG laser (532 nm, ≈50 mW), a polarizer, an analyzer, and a CCD camera. When *p*‐polarized light is directed onto the pure air–water interface at the Brewster angle (≈53.1°), zero reflectivity is observed. The presence of a monolayer causes light to be reflected because of the changed refractive index of the surface layer, which is then registered by the CCD camera after passing the analyzer. The lateral resolution of the BAM images was ≈2 μm.^44^, ^45^

**Infrared reflection absorption spectroscopy measurements (IRRAS)**: The experiments were performed using an IFS 66 FTIR spectrometer equipped with a liquid nitrogen cooled mercury cadmium telluride detector attached to an external air–water reflection unit (XA‐511, Bruker). The principle of the method and its application to Langmuir films at the air–water interfaces has been previously described.^46^, ^47^, ^48^, ^49^ A small reference trough and the larger sample trough are alternatively moved into the IR beam path by a shuttle system. The reflectance absorbance was calculated using −log(*R*/*R*_0_), with *R* being the reflectance of the sample and R_0_, the reflectance of the reference. The resolution and scanner speed in all experiments were 8 cm^−1^ and 20 kHz. The incident IR beam is polarized with a KRS‐5 wire grid polarizer. Spectra are co‐added over 200 scans for s‐polarized light and over 400 scans for p‐polarized light before being apodized using Blackman–Harris three‐term function and fast Fourier transformed after one level of zero filling.

**Grazing incidence X‐ray diffraction (GIXD) experiments**: The GIXD experiments were performed at the high resolution diffraction beamline P08 (PETRA III, DESY, Hamburg, Germany). For the measurements, a Langmuir trough was located in a hermetically sealed container with Kapton windows transparent for X‐rays. The trough was constantly flushed with helium (He) to avoid scattering of molecules from the air and to increase the signal to background ratio considerably. The synchrotron beam was monochromated by a set of two monochromators (silicon double crystal (Si111) and germanium double crystal (Ge311)). The photon energy was adjusted to 15 keV corresponding to a wavelength of 0.827 Å. Approximately 2 mm×50 mm of the monolayer surface were illuminated. The incident angle was adjusted to 0.07° to be below the critical angle for total external reflection for water. To decrease mechanically excited surface waves, a glass block was present in the subphase beneath the illuminated area of the monolayer. A Mythen (microstrip system for time resolved experiments) detector (DECTRIS, Baden, Switzerland) was rotated around the sample to detect the intensity of the diffracted beam as a function of the vertical scattering angle α_f_ and horizontal scattering angle 2θ. A Soller collimator (JJ X‐ray, Denmark) was located between the sample and the detector to restrict the in‐plane divergence of the diffracted beam.

Model peaks taken as Lorentzian in the in‐plane (Bragg peaks, *Q*_xy_) and Gaussian in the out‐of‐plane direction (Bragg rods, *Q*_z_) were fitted to the integrated data. Subsequently, the Bragg peak positions, respectively the centers of the Bragg rods are obtained and structure relevant information is gained. The presence of one Bragg peak located at the horizon describes a hexagonal lattice of non‐tilted lipid alkyl chains in a rotator phase, two peaks are typical for an orthorhombic and three peaks for an oblique lattice.^40^, ^50^, ^51^, ^52^, ^53^

**Total reflection X‐ray fluorescence (TRXF) experiments**: In recent years, TRXF has been established as an element‐specific complementary scattering technique.^38^, ^41^, ^54^ The TRXF measurements were carried out at beamline P08 (PETRA III, DESY, Hamburg, Germany) using the above described set‐up. The fluorescence signal was detected by an Amptek X‐123SDD detector (Amptek, Bedford, USA) placed almost parallel to the liquid surface and perpendicular to the photon beam axis. This detector position was chosen in order to keep the Compton scattering at the given polarization of the photons as low as possible. The footprint center of the incident beam was adjusted to the middle of the trough at the middle of the view angle of the fluorescence detector.^38^, ^40^

## Conflict of interest

*The authors declare no conflict of interest*.

## Supporting information

As a service to our authors and readers, this journal provides supporting information supplied by the authors. Such materials are peer reviewed and may be re‐organized for online delivery, but are not copy‐edited or typeset. Technical support issues arising from supporting information (other than missing files) should be addressed to the authors.

Supplementary
