# Supplementary material for: Thermodynamic and Structural Behavior of α‐Galactosylceramide and C6‐Functionalized α‐GalCer in 2D Layers at the Air–Liquid Interface
Source: Chembiochem. 2019 Nov 7;21(1-2):241–7. doi: 10.1002/cbic.201900491 (PMC7004034; doi:10.1002/cbic.201900491)
Supplement: Supplementary file 1 — Supplementary [file CBIC-21-241-s001.pdf]

CHEMBIOCHEM

Supporting Information

Thermodynamic and Structural Behavior of α-Galactosylceramide and C6-Functionalized α-GalCer in 2D Layers at the Air–Liquid Interface

Gerald Brezesinski,* Adam D. J. Calow, Claney L. Pereira, and Peter H. Seeberger[a]

cbic_201900491_sm_miscellaneous_information.pdf

Supporting information

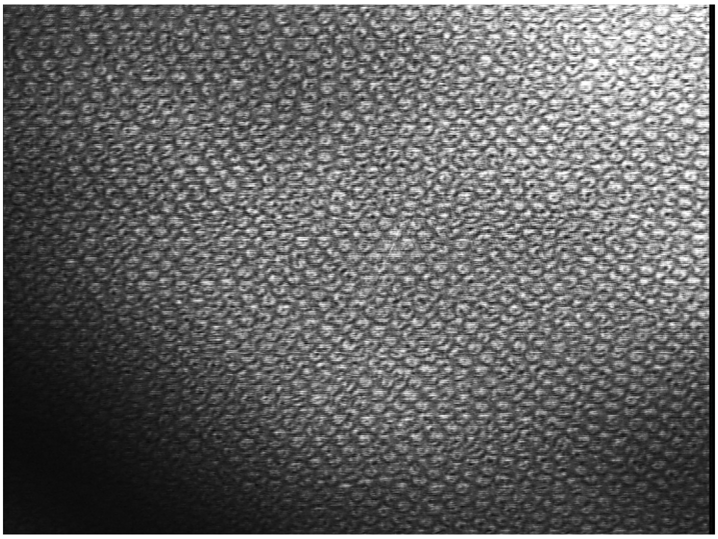

Figure S1. BAM image of the C6-functionalized α-GalCer **2** monolayer on pH 1 subphase at 20 °C after starting the nucleation during compression. The image has a size of 600 x 450 μm^2.

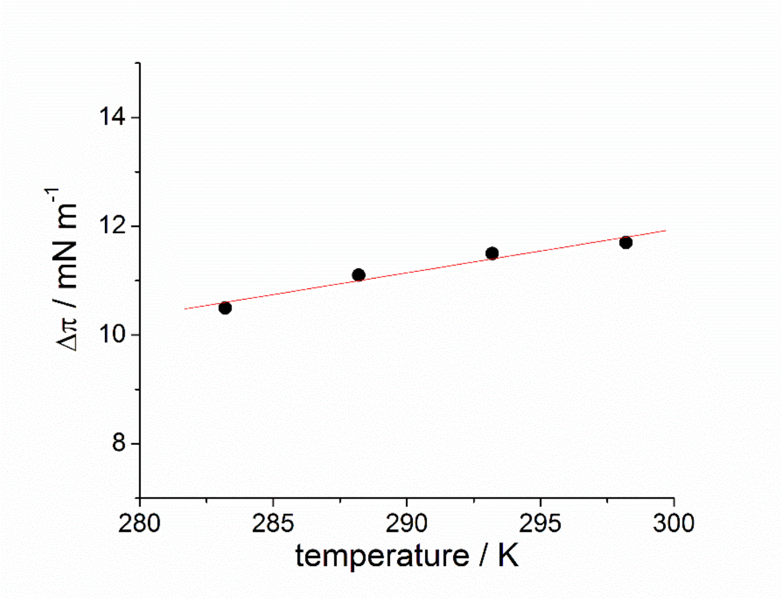

Figure S2. Pressure difference Δπ between the highest pressure needed for starting the nucleation and the corresponding transition pressure π_t in monolayers of the C6-functionalized α-GalCer **2** on pH 1 versus the temperature at which the isotherm has been measured.

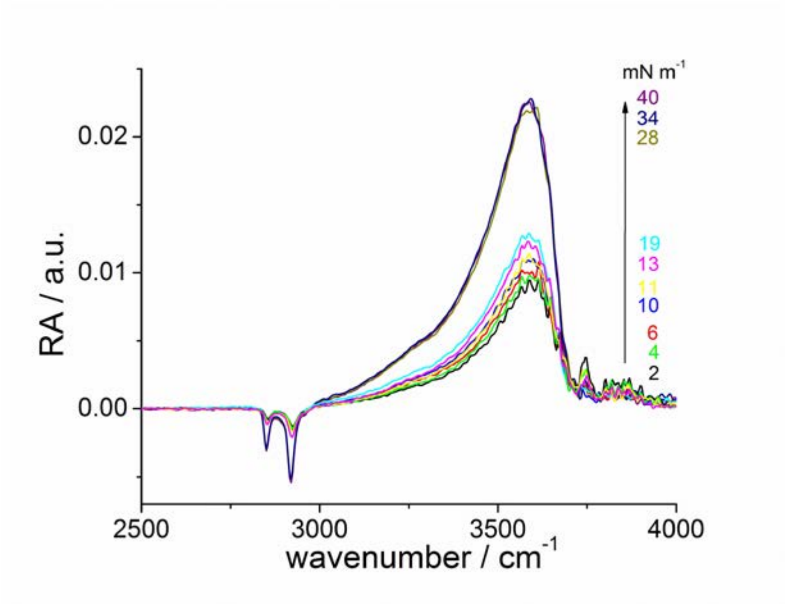

Figure S3. Part of the IRRA spectra of the C6-functionalized α-GalCer **2** monolayer on water at 20 °C taken along the isotherm at π = 2, 4, 6, 10, 11, 13, 19, 28, 34, and 40 mN·m^{-1}. The spectra are recorded using p-polarized light and an angle of incidence of 40°.

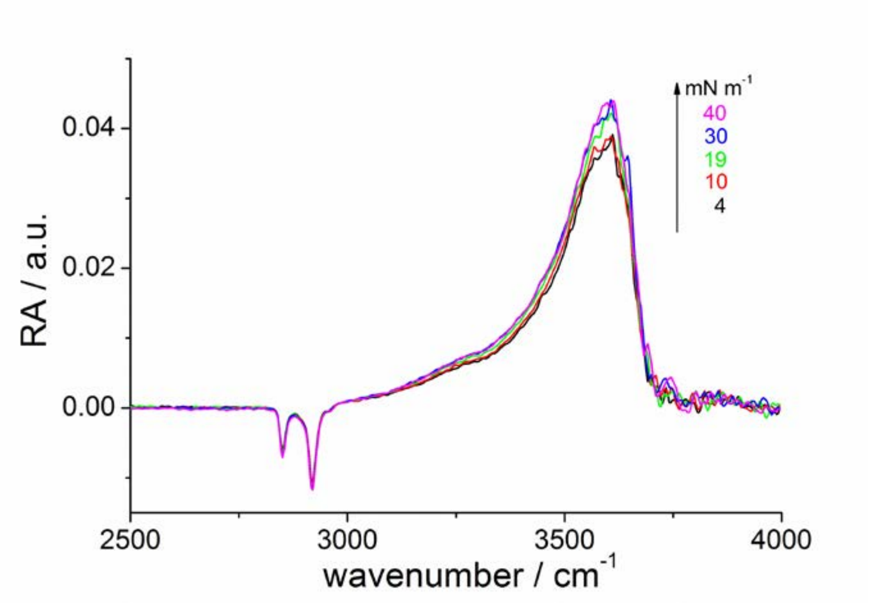

Figure S4. Part of the IRRA spectra of KRN7000 **1** monolayer on water at 20 °C taken along the isotherm at π = 4, 10, 19, 30, and 40 mN·m^{-1}. The spectra are recorded using p-polarized light and an angle of incidence of 40°.

Synthesis of C6-Functionalized α-GalCer 2

The numbering in the experimental section refers to the scheme below and is consistent with the numbering throughout the manuscript.

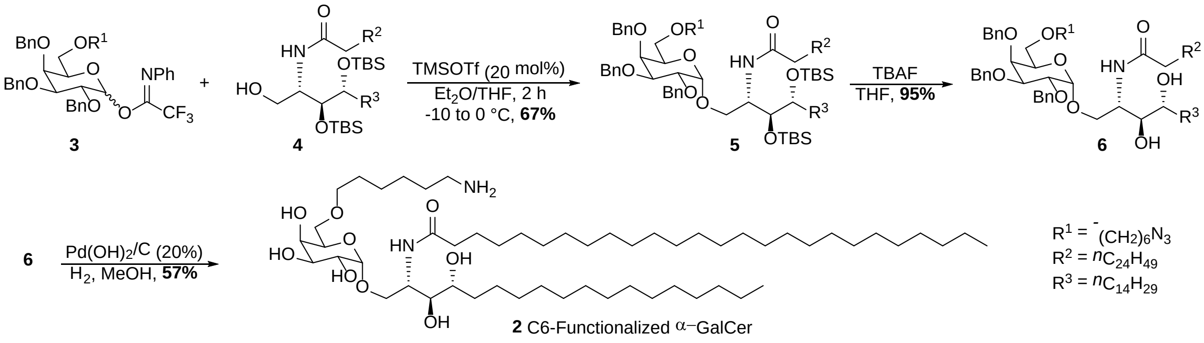

Commercial grade solvents were used unless stated otherwise. Dry solvents were obtained from a Waters Dry Solvent System. Solvents for chromatography were distilled prior to use. Sensitive reactions were carried out in heat-dried glassware and under an argon atmosphere. Analytical thin layer chromatography (TLC) was performed on Kieselgel 60 F254 glass plates precoated with a 0.25 mm thickness of silica gel. Spots were visualized by staining with vanillin solution (6% (w/v) vanillin and 10% (v/v) sulfuric acid in 95% EtOH) or Hanessian's stain (5% (w/v) ammonium molybdate, 1% (w/v) cerium(II) sulfate and 10% (v/v) sulfuric acid in water). Silica column chromatography was performed on Fluka Kieselgel 60 (230-400 mesh).

^{1}H, ^{13}C and two-dimensional NMR spectra were measured with a Varian 400-MR, 600-MR and Bruker Avance 700 spectrometer at 296 K. Chemical shifts (δ) are reported in parts per million (ppm) relative to the respective residual solvent peaks ($CDCl_3$: δ 7.27 in ^{1}H and 77.23 in ^{13}C NMR; CD_3OD: δ 3.31 in ^{1}H and 49.15 in ^{13}C NMR; D_2O: δ 4.80 in ^{1}H NMR; Acetone-d_6: δ 2.05 in ^{1}H and 29.92 in ^{13}C NMR).The following abbreviations are used to indicate peak multiplicities: *s* singlet; *d* doublet; *dd* doublet of doublets; *t* triplet; *dt* doublet of triplets; *q* quartet; *m* multiplet. Coupling constants (*J*) are reported in Hertz (Hz). Optical rotation (OR) measurements were carried out with a Schmidt & Haensch UniPol L1000 polarimeter at λ = 589 nm and a concentration (c) expressed in g/100 mL in the solvent noted in parentheses. High resolution mass spectrometry (HRMS) was performed at the Free University Berlin, Mass Spectrometry Core Facility, with an Agilent 6210 ESI-TOF mass spectrometer. Infrared (IR) spectra were measured with a Perkin Elmer 100 FTIR spectrometer.

The synthesis of C6-modified α-GalCer **2** was prepared previously by this group.[1] The key modified steps are shown below:

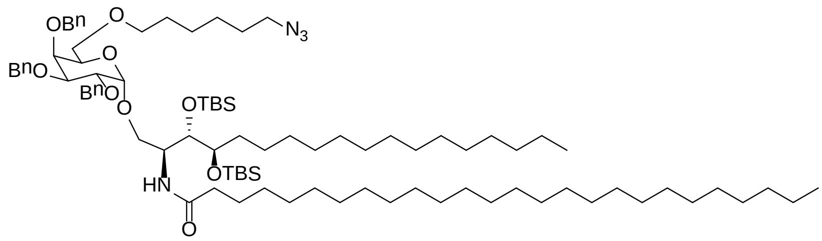

Imidate **3** (591 mg, 0.79 mmol) and ceramide **4** (610 mg, 0.66 mmol) were combined in toluene and concentrated *in vacuo* 3 times to facilitate the removal of water. After 3 h under high vacuum (<0.5 mBar), the resulting oil was diluted in anhydrous diethyl ether (6 mL) and anhydrous tetrahydrofuran (1.2 mL). Dried acid washed 4 Å molecular sieve pellets were added and the solution was stirred for 1 h at -10 ºC. Trimethylsilyl trifluoromethanesulfonate (18 µL, 0.10 mmol) was added and the reaction was allowed to warm to 0 ºC over 2.5 h. The reaction was quenched by the slow addition of triethylamine (1 mL) and the solution was concentrated *in vacuo*. Purification by flash silica gel chromatography (hexane : EtOAc, 8:1 → hexane : EtAcO, 3:1) allowed for the isolation of the protected C6-modified α-GalCer **5** as a viscous colourless oil (653 mg, 67%). All physical and spectroscopic properties were identical to those reported in the literature.[1]

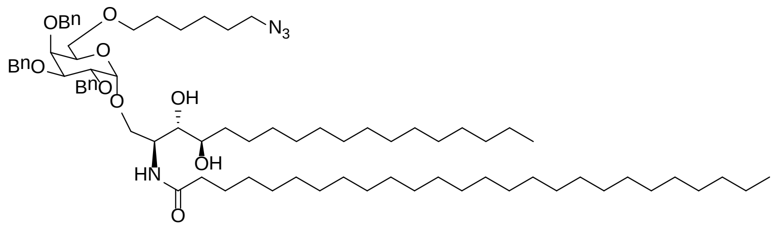

To a flask containing C6-modified α-GalCer **5** (61 mg, 41 µmol) was added anhydrous tetrohydrofuran (2 mL) and the solution was cooled to 0 ºC under nitrogen. To the solution was added tetrabutylammonium fluoride in THF (0.41 mL, 1 M) dropwise over 1 min. After 3 h the solution was concentrated under vacuum to *ca.* 0.5 mL. Purification by silica gel chromatography (hexane:EtOAc, 2:1 → hexane:EtAcO, 1:1) allowed for the isolation of the desilylated α-GalCer **6** as a viscous colourless oil (49 mg, 95%). All physical and spectroscopic properties were identical to those reported in the literature.[1]

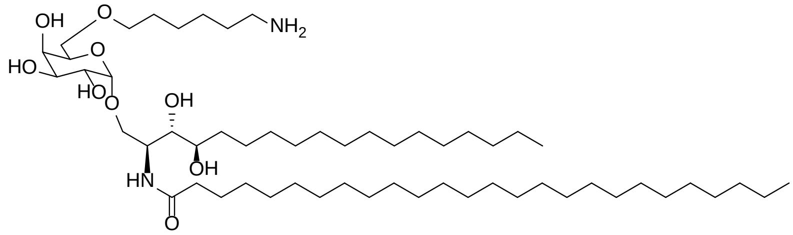

To the desilylated α-GalCer **6** (71 mg, 57 µmol) was added methanol (0.8 mL) and chloroform (0.2 mL). Palladium(II) hydroxide on carbon (14 mg) was added and the solution

was purged of oxygen using a vacuum-nitrogen cycle followed by 3 vacuum-hydrogen cycles. The resulting suspension was stirred over a hydrogenous atmosphere for 16 h. Filtration through celite and concentration *in vacuo* resulted in the formation of a yellow solid. Purification by silica gel chromatography (DCM → DCM:MeOH, 4:1) allowed for the isolation of C6-Functionalized α-GalCer **2** as a pale yellow solid (31 mg, 57%); ^{1}H NMR (400 MHz, *pyridine*-d5) δ 8.86 (d, J = 8.6 Hz, 1H), 5.61 (d, $J_{αC-H}$ = 3.8 Hz, 1H), 5.32 – 5.25 (m, 1H), 4.73 – 4.69 (m, 1H), 4.66 – 4.63 (m, 1H), 4.51 – 4.39 (m, 3H), 4.39 – 4.36 (m, 3H), 4.08 – 4.01 (m, 2H), 3.52 – 3.47 (m, 2H), 3.34 – 3.30 (m, 2H), 2.59 – 2.54 (m, 2H), 2.35 – 2.26 (m, 1H), 2.02 – 1.97 (m, 2H), 1.95 – 1.82 (m, 4H), 1.76 – 1.63 (m, 1H), 1.56 – 1.42 (m, 7H), 1.33 – 1.26 (m, 66H), 0.89 – 0.86 (m, 6H); All physical and sprectroscopic properties were identical to those reported in the literature.[1]

Reference

1. M. Cavallari, P. Stallforth, A. Kalinichenko, D. C. K. Rathwell, T. M. A. Gronewold, A. Adibekian, L. Mori, R. Landmann, P. H. Seeberger and G. De Libero, *Nat. Chem. Biol.* **2014**, *10,* 950-956.
